# Identification of a Protein Phosphatase-1/Phospholamban Complex That Is Regulated by cAMP-Dependent Phosphorylation

**DOI:** 10.1371/journal.pone.0080867

**Published:** 2013-11-14

**Authors:** Elizabeth Vafiadaki, Demetrios A. Arvanitis, Despina Sanoudou, Evangelia G. Kranias

**Affiliations:** 1 Molecular Biology Division, Biomedical Research Foundation, Academy of Athens, Athens, Greece; 2 Department of Pharmacology, Medical School, University of Athens, Greece; 3 Department of Pharmacology and Cell Biophysics, University of Cincinnati College of Medicine, Cincinnati, Ohio, United States of America; University of Florence, Italy

## Abstract

In human and experimental heart failure, the activity of the type 1 phosphatase is significantly increased, associated with dephosphorylation of phospholamban, inhibition of the sarco(endo)plasmic reticulum Ca^2+^ transport ATPase (SERCA2a) and depressed function. In the current study, we investigated the molecular mechanisms controlling protein phosphatase-1 activity. Using recombinant proteins and complementary *in vitro* binding studies, we identified a multi-protein complex centered on protein phosphatase-1 that includes its muscle specific glycogen-targeting subunit G_M_ and substrate phospholamban. G_M_ interacts directly with phospholamban and this association is mediated by the cytosolic regions of the proteins. Our findings suggest the involvement of G_M_ in mediating formation of the phosphatase-1/G_M_/phospholamban complex through the direct and independent interactions of G_M_ with both protein phosphatase-1 and phospholamban. Importantly, the protein phosphatase-1/G_M_/phospholamban complex dissociates upon protein kinase A phosphorylation, indicating its significance in the β-adrenergic signalling axis. Moreover, protein phosphatase-1 activity is regulated by two binding partners, inhibitor-1 and the small heat shock protein 20, Hsp20. Indeed, human genetic variants of inhibitor-1 (G147D) or Hsp20 (P20L) result in reduced binding and inhibition of protein phosphatase-1, suggesting aberrant enzymatic regulation in human carriers. These findings provide insights into the mechanisms underlying fine-tuned regulation of protein phosphatase-1 and its impact on the SERCA2/phospholamban interactome in cardiac function.

## Introduction

Protein phosphorylation is tightly regulated by the intricate balance between protein kinase and phosphatase activities, which influence various cellular pathways and their responses to extracellular signals. In cardiac muscle, the type 1 protein phosphatase (PP1) plays a critical role as a regulator of calcium cycling and contractility as well as the heart's responses to β-adrenergic stimulation [1]. These effects of PP1 are partially attributed to dephosphorylation of phospholamban (PLN), the reversible regulator of the sarco(endo)plasmic reticulum (SR) Ca^2+^ transport ATPase (SERCA2a), impacting cardiac performance [2]. Dephosphorylated PLN is an inhibitor of SERCA2a’s affinity for Ca^2+^, while β-adrenergic stimulation leads to PLN phosphorylation by cAMP-dependent protein kinase (PKA) and Ca^2+^-calmodulin-dependent protein kinase (CaMKII), relieving SERCA2a inhibition and enhancing SR Ca^2+^ transport as well as cardiac relaxation. The significance of PLN phosphorylation at serine 16 (Ser16) by PKA and threonine 17 (Thr 17) by CaMKII has been demonstrated through the detailed characterization of PLN-mutant mouse models [3], [4], [5], [6]. Importantly, the phosphorylation levels of PLN at Ser16 and Thr17 are decreased in human failing hearts [7], [8], [9], due to increased PP1 activity [8] and this has been suggested to contribute to cardiac dysfunction. Indeed, transgenic overexpression of PP1 in the mouse heart at similar levels as human failing hearts resulted in depressed contractility, heart failure and early death [10].

PP1 is a holoenzyme that consists of the catalytic subunit, which possesses enzymatic activity, and regulatory subunits that are implicated in defining substrate specificity and modulating catalytic activity [11]. In cardiac muscle, PP1 is regulated by the endogenous proteins, inhibitor-1 and inhibitor-2, while our recent findings have uncovered a role for the small heat shock protein 20 (Hsp20) as a novel regulator of PP1 in the heart [12], [13]. Genetic manipulation of these inhibitory molecules has demonstrated their functional significance in the control of PP1 activity, SR Ca^2+^ cycling and cardiac contractility [10], [13], [14], [15]. In addition, decreased levels and activity of inhibitor-1 have been correlated with PLN dephosphorylation and depressed Ca^2+^ cycling in failing human hearts [8], while Hsp20 protein and phosphorylation levels have been observed to increase under similar conditions [16], [17]. These findings denote the critical role of PP1 and its auxiliary proteins in regulation of PLN activity and cardiac function.

While the impact of PP1 on PLN dephosphorylation and SR Ca^2+^ cycling has been well established, the molecular mechanisms underlying this process have not yet been widely explored. In the current study, we identified a multi-meric PP1 protein complex, composed of the PP1 targeting subunit G_M_, substrate PLN and two PP1 binding partners, inhibitor-1 and Hsp20. This PP1-interactome is regulated by PKA phosphorylation, highlighting its significance in the β-adrenergic signalling axis under physiological and stress conditions.

## Materials and Methods

### Generation of recombinant proteins

The conditions used for the generation of the maltose binding protein (MBP) constructs containing full length MBP-PP1 (aa1-330) as well as the overlapping fragments MBP-PP1 (amino acid 1-187) and MBP-PP1 (amino acid 163-330) have been previously described [13]. To generate the G_M_ expression constructs, RT-PCR was performed on human muscle cDNA using primers 5′ CCAATGGAGCCTTCTGAAGTA 3′ and 5′ TCCCTTTACGGAGCTTTCTG 3′ for G_M_ (amino acids 1-386), primers 5′ AGCTCCGTAAAGGGAGATTTTT 3′ and 5′ TCATGTGGATCAAACGCTGT 3′for G_M_ (amino acids 382-778) and primers 5′ CAAGAGACAGCACGAAGTGA 3′ and 5′ TACTTCTTTTTGACAGACTCTTTTTG 3′ for G_M_ (amino acids 382-778) construct. PCR products were cloned in the EcoRI/SalI sites of the pGEX5x-1 (Amersham Biosciences, Uppsala, Sweden) and pMALc2x (New Englands Biolabs, Ipswich, USA). The authenticity of all constructs was confirmed by sequence analysis (Macrogen Inc). The GST-PLN (amino acids 1–37) construct containing the cytoplasmic region of the protein has been previously described [18] and the conditions for the generation of the HAX-1 construct encoding amino acids 118-260 have also been reported [19].

Protein expression of the above constructs was performed as previously described [18]. Recombinant proteins were purified by affinity chromatography on Glutathione Sepharose^™^ 4B Beads (Amersham Biosciences) or amylose resin (New England Biolabs), according to manufacturer's instructions. Fusion-peptides were eluted from the beads in accordance with manufacturer's instructions.

### Generation of recombinant proteins for Inhibitor-1 and Hsp20 human variants

The inhibitor-1 plasmids containing wild type or G147D variant sequences [20] were used as templates for PCR amplification using primers 5′ GCCATGGAGCAAGACAACA 3′ and 5′ CCTCTCTCAGACCGAGTTGG 3′. The resulting PCR products were cloned in the EcoRI/SalI sites of the pGEX5-x1 vector and protein expression was performed as described above.

The generation of the full length Hsp20 construct has been previously described [13]. The plasmid containing the Hsp20-P20L variant [21] was used as template for PCR amplification using primers 5′AGCAGGATGGAGATCCCTGT 3′ and 5′ CCAGCCCCCTCCTACTTG 3′. The resulting PCR products were cloned in the EcoRI/SalI sites of the pGEX5-x1 vector and protein expression was performed as described above.

### GST pull down assay

Pull down assays were performed as previously described [18]. Briefly, cardiac homogenates of mouse origin were prepared in 10 mM NaPO_4_ (pH 7.2), 2 mM EDTA, 10 mM NaN_3_, 120 mM NaCl and 1% NP-40, supplemented with protease inhibitors (Sigma-Aldrich, Munich, Germany). Equivalent amounts of recombinant GST and GST-PP1 (amino acids 1-330) recombinant proteins bound to glutathione-Sepharose 4B^™^ resin (Amersham Biosciences) were mixed with 0.5 mg of cardiac homogenates at 4°C for 16 h. The beads were washed with 10 mM NaPO_4_ (pH 7.2), 10 mM NaN_3_, 120 mM NaCl, 0.1% (v/v) Tween-20 and were subsequently analyzed by western blot using PLN primary antibody (Affinity Bioreagents, Golden, USA) and peroxidase-conjugated anti-mouse (Sigma-Aldrich,) secondary antibodies. Immunoreactive bands were detected using Pierce ECL Plus reagents (Thermo Scientific, Rockford, USA).

In another set of experiments, pull down assays were performed using equivalent amount of GST and GST- G_M_ (aa382-778) or GST and GST- G_M_ (aa1-386) recombinant proteins bound to glutathione-Sepharose 4B^™^ resin (Amersham Biosciences) and cardiac muscle homogenates as described above. Samples were then analyzed by western blot using PP1 (Santa Cruz Biotechnology, Inc, Dallas, USA) or PLN primary antibodies. Similarly, pull down assays were performed using GST, GST- G_M_ (aa1-778) or GST-Inhibitor-1 recombinant proteins and cardiac muscle homogenates as described above. Samples were then analyzed by western blot using PP1 (Santa Cruz Biotechnology, Inc), or PLN primary antibodies.

### Blot overlay assay

Protein interactions were evaluated *in vitro* by blot overlay assays, as previously described [13], [22]. Briefly, purified MBP, MBP-PP1 (amino acids 1−330), MBP-PP1 (amino acid 1-187) and MBP-PP1 (amino acid 163-330) and MBP-HAX-1 (amino acid 118-260) recombinant proteins were separated by SDS-PAGE and transferred to nitrocellulose membranes. Retention of the MBP-tag in the fusion proteins enabled the use of the MBP protein, encoded by the empty vector, as a negative control in these binding assays. Following blocking, the membranes were incubated with GST-PLN fusion protein. The blots were probed with anti-GST and the immunoreactive bands were visualized using ECL reagents. In another set of experiments, MBP, MBP-G_M_ (amino acids 1−386), MBP-G_M_ (amino acids 382−778) and MBP-G_M_ (amino acids 756−1222) recombinant proteins were allowed to interact with either GST-PLN or GST-PP1 (amino acids 1−330) proteins as described above. Blot overlay assays were also performed to assess protein binding between MBP-PP1 (amino acids 1−330) and GST-PLN (amino acids 1-37) in the absence or presence of GST- G_M_ (aa1-778). For this analysis, MBP-PP1 (amino acids 1−330) recombinant protein was separated by SDS-PAGE and was overlaid with GST-PLN alone or in the presence of GST- G_M_ (aa1-778). Protein binding was determined by western blot analysis using the PLN antibody.

In a different set of experiments, equal amounts of GST-Inhibitor-1 or GST-Hsp20 proteins were separated by SDS-PAGE and were allowed to interact with MBP-PP1 (amino acids 1−330) recombinant protein, as described above. Western blot analysis with MBP antibody (New England Biolabs) determined changes in the level of PP1 binding to wild type or human variant proteins.

### Protein Kinase A phosphorylation

Protein phosphorylation was performed *in vitro* using cAMP-dependent Protein Kinase (PKA), catalytic subunit (New England Biolabs), according to manufacturer's instructions. Briefly, cardiac homogenates or recombinant proteins were incubated with 1X PKA Reaction Buffer (50 mM Tris-HCl pH 7.5, 10 mM MgCl_2_)_,_ supplemented with 200 µM ATP (Sigma-Aldrich) and 1,250 units of PKA catalytic subunit. Samples were incubated at 30°C for 1 hour and were subsequently used for pull downs, blot overlay assays or PP1 activity measurements. PLN phosphorylation levels were detected by western blot analysis using the phospho-PLN (Ser16) antibody (Upstate, New York, USA) while phosphorylation levels of GST-Inhibitor-1 or GST-Hsp20 recombinant proteins were determined using the phospho-I-1 (Thr35) antibody (Santa Cruz Biotechnology, Inc) or phospho-Hsp20 (Ser16) antibody (AbCam, Cambridge, UK) respectively.

### PP1 activity measurements

Protein phosphatase activity was evaluated using PP1 enzyme (New England Biolabs), in accordance to manufacturer's instructions. Reactions were performed using recombinant proteins in the presence of 1X Reaction Buffer (50 mM HEPES pH 7.5, 100 mM NaCl, 2 mM DTT, 0.01% Brij 35), supplemented with 1 mM MnCl_2_. Following the addition of 50 mM p-Nitrophenyl Phosphate substrate (New England Biolabs) and 1.25 units of PP1, samples were incubated at 30°C for 10 minutes. Reactions were then terminated by addition of 0.5 M EDTA and the activity of PP1 on p-Nitrophenyl Phosphate substrate was determined by spectrophotometric measurement at 405 nm.

## Results

### PP1 association with PLN and effect of PKA

**Figure 1:**
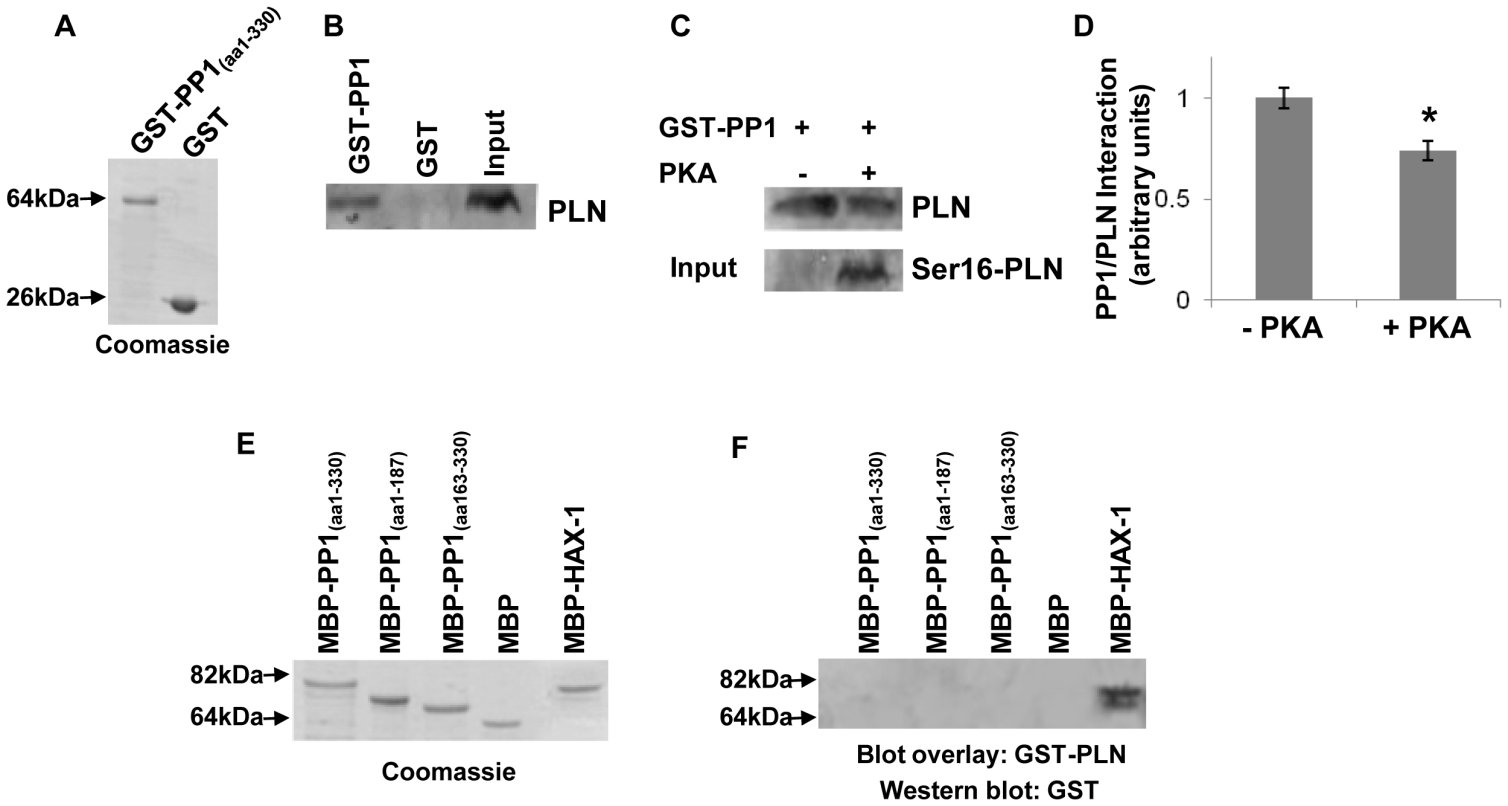
PP1 associates with PLN through an indirect interaction that is inhibited by PLN phosphorylation. (A) Coomassie blue staining of purified GST-PP1 and GST recombinant proteins. (B) Western blot analysis of pull down assays revealed the interaction of PP1 with PLN. (C) Pull down assays using phosphorylated protein homogenates indicated that Ser16-PLN phosphorylation results in reduced association of PLN with PP1. (D) Quantification of PP1 and PLN association showed a significant reduction upon PKA phosphorylation. n = 4; *t*-test, two-tailed, *P<0.05 vs –PKA. (E) Coomassie blue staining of purified MBP, MBP-PP1 and MBP-HAX-1 proteins. (F) Blot overlay assays using GST-PLN determined the absence of direct binding to PP1.

As PP1 is the main enzyme responsible for PLN dephosphorylation, we sought to investigate the molecular mechanisms underlying this regulation and whether it may be mediated through a direct interaction between the two proteins. To test this hypothesis, we performed pull down assays in cardiac homogenates, using full length GST-PP1 (aa1-330) or GST recombinant proteins (Figure 1A). Western blot analysis of pull down samples determined the presence of native PLN protein in the GST-PP1 but not the GST control sample (Figure 1B), a finding that reveals the association of PP1 with the PLN/SERCA protein complex. Given the significance of PLN phosphorylation and the participation of PP1 in modulation of β-adrenergic signaling of the heart, we next evaluated whether this protein complex may be regulated by PKA phosphorylation. For this analysis, we used cardiac homogenates that had been previously treated with PKA and GST-PP1 protein for pull down assays. A sample containing equal amounts of GST-PP1 recombinant protein and non-PKA phosphorylated cardiac homogenates was used as control in these assays. PLN phosphorylation was confirmed with an antibody recognizing specifically the phosphorylated Ser16 residue. Western blot analysis of the pull down samples indicated a reduction in PLN binding to GST-PP1 in the PKA-treated sample, compared to the non-phosphorylated sample (Figure 1C). Quantitative analysis from three independent experiments showed that PLN phosphorylation led to ∼25% reduction in its binding to PP1 (Figure 1D, n = 4; *P*<0.05). This finding implicates the PP1/PLN interaction in the β-adrenergic response, hence indicating its physiological significance in the heart.

Having determined the existence of the PP1/PLN protein complex, we next sought to investigate whether this protein association occurs through a direct interaction between PP1 and PLN. To examine this, we performed in vitro binding assays using recombinant PP1 and PLN proteins. In particular, blot overlay assays were carried out using GST-PLN (aa1-37) that contains the cytoplasmic region of the protein, and either full length MBP-PP1 (aa1-330) or two overlapping fragments of PP1 (aa1-187 and aa163-330, respectively) (Figure 1E). Western blot analysis revealed that PLN did not bind to any of the MBP-PP1 proteins examined, therefore indicating the absence of direct interaction between PLN and PP1 (Figure 1F). Recombinant MBP-HAX-1, a previously identified direct binding partner of PLN [18], was included as a positive control in these reactions (Figure 1F). Given the existence of the PP1/PLN complex in cardiac homogenates, the absence of binding between PP1 and PLN in this in vitro binding system, points towards the presence of another protein partner, which could facilitate the formation of the PP1/PLN protein complex.

### G_M_, a targeting subunit of PP1, interacts directly with both PLN and PP1

**Figure 2:**
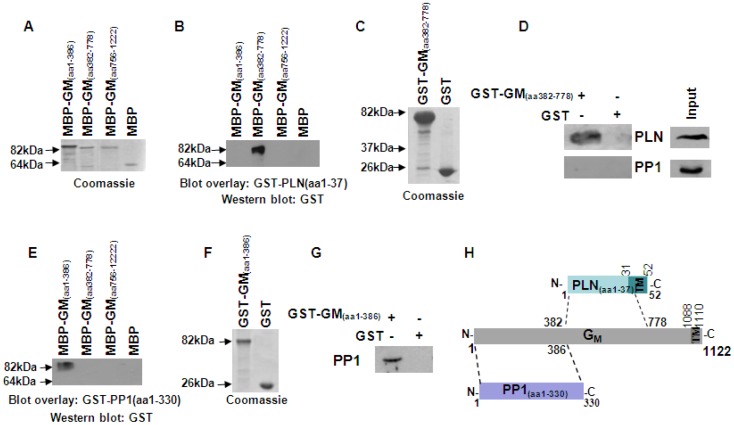
G_M_ interacts directly and independently with both PLN and PP1. (A) Coomassie blue stained gel showing purified MBP-G_M_ recombinant proteins. Blot overlay assays with (B) GST-PLN or (E) GST-PP1 determined that different fragments of G_M_ interact directly with either PLN or PP1. (C) Coomassie blue stained gel showing purified GST and GST-G_M_ (amino acid 382-778) recombinant proteins. (D) Western blot analysis of pull down assays using GST-G_M_ (amino acid 382-778) demonstrated the association of this fragment with PLN but not PP1 in cardiac homogenates. (F) Coomassie blue stained gel showing purified GST and GST-G_M_ (amino acid 1-386) recombinant proteins. (G) Western blot analysis of pull down assays using GST-G_M_ (amino acid 1-386) confirmed binding of PP1 to this G_M_ fragment in cardiac homogenates. (H) Diagrammatic representation of G_M_, illustrating the different protein regions involved in PP1 or PLN binding. The transmembrane domain (TM), predicted by SMART analysis [58] of G_M_ protein sequence, is also shown.

A previous study has suggested that PLN may interact with G_M_, the muscle-specific glycogen-targeting subunit of PP1 [23]. To investigate this, we generated several constructs that enabled us to produce overlapping fragments of recombinant MBP-G_M_ protein. Since G_M_ is a large protein (∼125 kDa in size), it was not feasible to generate a full length construct for recombinant protein production in bacterial expression systems. Instead, we chose to generate three constructs, containing overlapping fragments of G_M_ that included amino acids 1-386, 382-778 and 756-1222 (Figure 2A). Blot overlay assays on these MBP-G_M_ proteins with GST-PLN recombinant protein demonstrated a direct interaction between the two proteins and showed that the region of G_M_ that is required for its interaction with PLN includes amino acids 382-778 (Figure 2B). To confirm this finding, we generated a GST-G_M_ recombinant protein, that contained amino acids 382-778 (Figure 2C), and used it in pull down assays of cardiac homogenates. Western blot analysis revealed the presence of PLN in the GST-G_M_ but not the GST control sample, supporting the involvement of this G_M_ fragment in its association with PLN (Figure 2D).

Having determined the interaction between G_M_ and PLN, we next examined the association of G_M_ with PP1 in our in vitro experimental system. To assess this, we performed blot overlay assays using the three MBP-G_M_ overlapping fragments containing amino acids 1-386, 382-778 or 756-1222 and full length GST-PP1 recombinant protein. Western blot analysis determined the direct binding of PP1 to G_M_, which involved the N-terminal fragment of G_M_ that includes amino acids 1-386 (Figure 2E). The specificity of this region in mediating the PP1/G_M_ interaction was confirmed by pull down assays in cardiac homogenates, using GST or GST-G_M_ that contained amino acids 1-386 (Figure 2F), followed by the detection of PP1 in the GST-G_M_ sample (Figure 2G). Further support towards this was provided by the absence of PP1 in the G_M_ pull down sample, which only contained the G_M_ region that is involved in PLN binding (Figure 2D). Collectively, our findings indicate the direct and independent interaction of G_M_ with both PP1 and PLN, pointing towards its possible role in mediating the formation of the PP1/PLN protein complex. The N-terminal region of G_M_ (amino acids 1-386) is involved in PP1 targeting, while a more central fragment of G_M_ (amino acids 382-778) associates with PLN (Figure 2H).

### PKA phosphorylation inhibits the formation of G_M_/PLN and G_M_/PP1 protein complexes

**Figure 3:**
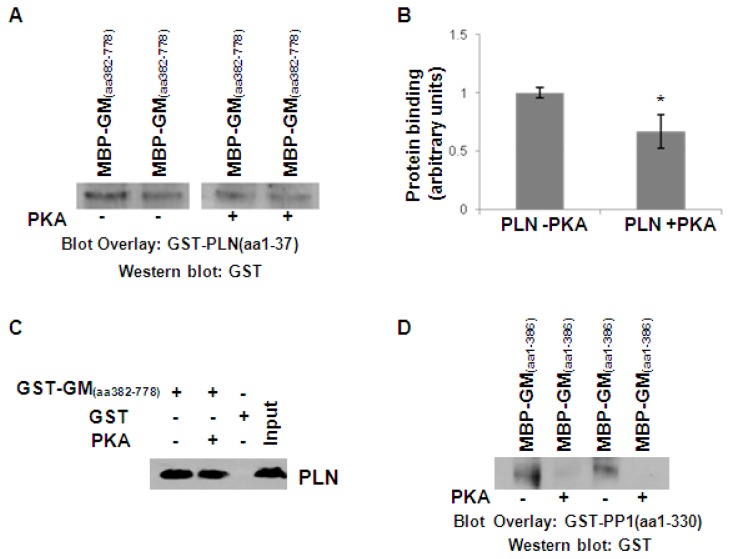
G_M_ phosphorylation inhibits its interactions with PLN and PP1. (A) Blot overlay assays on MBP-G_M_ (amino acid 382-778) recombinant protein was performed using non-phosphorylated or PKA-phosphorylated GST-PLN protein. Western blot analysis revealed decreased binding of phosphorylated PLN to G_M_. (B) Quantification of G_M_ and PLN binding established a significant reduction upon PLN phosphorylation. n = 6; *t*-test, two-tailed, *P<0.05 vs –PKA. (C) Pull down assays using GST-G_M_ (amino acid 382-778) recombinant protein and phosphorylated or non-phosphorylated cardiac homogenates illustrated the reduced association of PLN with G_M_ in the PKA-phosphorylated sample. (D) Blot overlay using phosphorylated or non-phosphorylated MBP-G_M_ (amino acid 1-386) revealed the lack of PP1 binding to phosphorylated G_M_ protein.

PLN phosphorylation has been shown to affect its interactions with SERCA2 [24] as well as its new partner HAX-1 [18]. Thus, we next sought to investigate whether phosphorylation of PLN may also regulate its binding with G_M_. To test this, we performed blot overlay assays using GST-PLN recombinant protein that had been previously phosphorylated by PKA. In parallel, blot overlay assays were performed using non-phosphorylated GST-PLN protein, which served as a control. Evaluation of phosphorylated PLN binding to G_M_, in comparison to non-phosphorylated protein association, revealed a significant decrease in this interaction upon PLN phosphorylation (Figure 3A-B, n = 6; *P*<0.05). Inhibition of G_M_ binding upon PLN phosphorylation was also confirmed by pull down assays, using GST-G_M_ recombinant protein containing amino acids 382-778 and cardiac homogenates that had been previously treated with PKA (Figure 3C). Co-immunoprecipitation assays in PKA or non-PKA treated cardiac homogenates also supported the reduced association of PLN/G_M_ upon protein phosphorylation (data not shown).

In addition to PLN, G_M_ also represents a target for PKA phosphorylation and it has been shown that Ser67 represents a critical residue for this modification [25]. Since Ser67 is located within the N-terminal fragment of G_M_ that binds to PP1, we performed blot overlay assays to evaluate the effect of phosphorylation on PP1/G_M_ association. For this analysis, phosphorylated or non-phosphorylated MBP- G_M_ protein (aa1-386) was used. Western blots indicated that there was no detectable binding of phosphorylated G_M_ recombinant protein to PP1 (Figure 3D). Collectively, these findings point out the critical role of protein phosphorylation in modulating PP1 protein complexes with PLN phosphorylation attenuating its interaction with G_M_ and G_M_ phosphorylation leading to its dissociation from PP1. Combined with the results obtained from the pull down assays in phosphorylated cardiac homogenates, it becomes evident that PKA phosphorylation represents an important regulatory mechanism, inhibiting PP1/G_M_/PLN interactions.

### G_M_ is required for effective PP1 targeting to its substrate PLN at the SR

**Figure 4:**
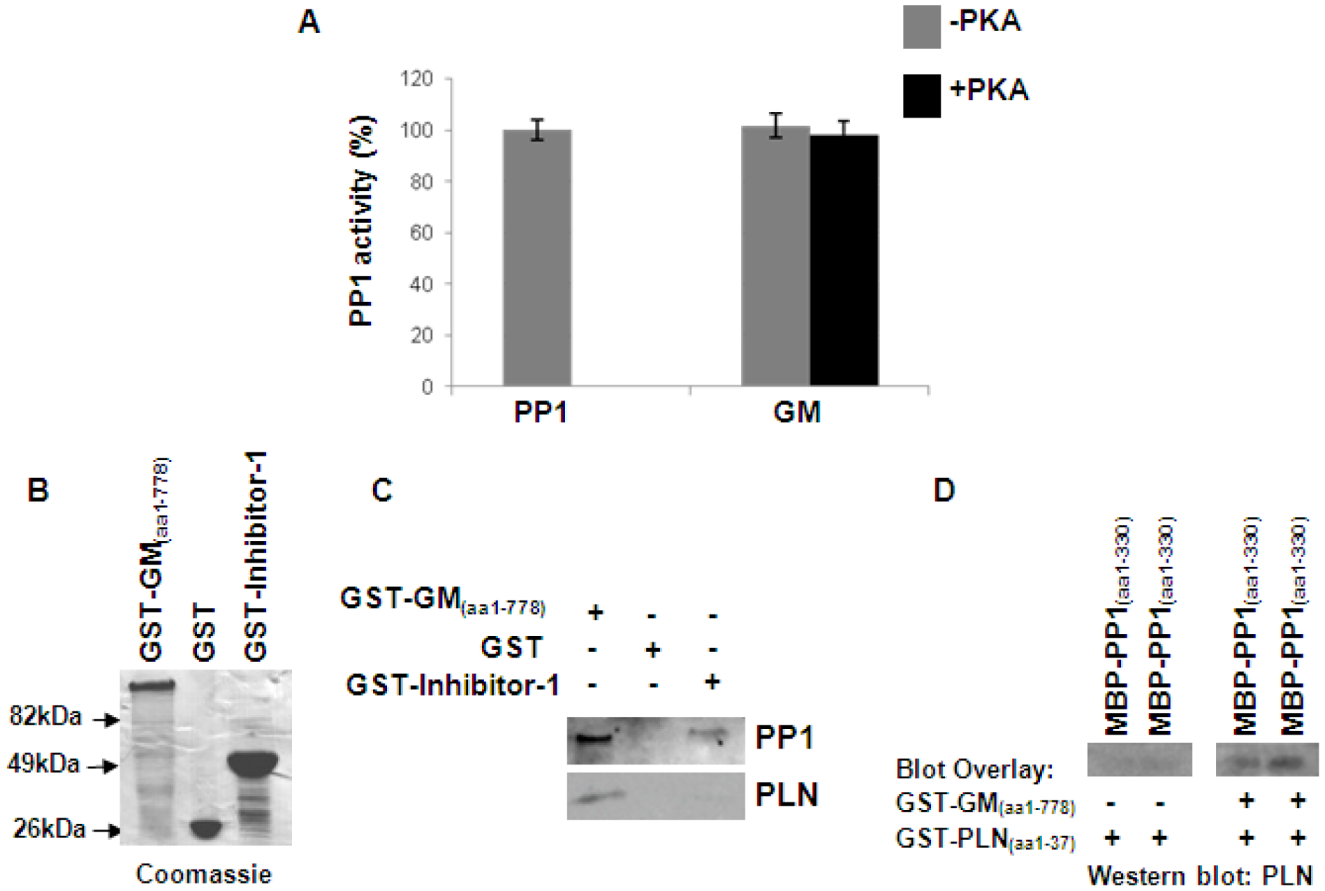
G_M_ is required for effective targeting of PP1 to its substrate PLN. (A) PP1 activity measurements showed that the presence of G_M_ does not affect PP1 activity. (B) Purified GST-G_M_ (amino acid 1-778), GST-Inhibitor-1 or GST proteins were used in pull down assays performed in cardiac homogenates. (C) Western blot analysis demonstrated the preferential association of PP1 with PLN in the GST-G_M_ sample, indicating the requirement of G_M_ for effective PP1 targeting to its substrate. (D) Blot overlay assays between MBP-PP1 and GST-PLN recombinant proteins were performed in the absence or presence of GST-G_M_. Immunodetection with the PLN antibody indicated protein association that occurred only in the presence of G_M_, suggesting the involvement of G_M_ in mediating the formation of the PP1/G_M_/PLN complex.

It is believed that regulatory subunits of PP1 function either as targeting subunits, guiding PP1 to the desired subcellular location and its substrate, or as components regulating its catalytic activity [11]. In order to further investigate the functional significance of the PP1/G_M_ complex in the heart, we evaluated the effect of G_M_ on PP1 activity. Interestingly, the presence of G_M_ did not affect the activity of PP1 (Figure 4A), suggesting that the PP1/G_M_ interaction does not influence its catalytic activity but may modulate PP1's subcellular targeting and substrate specificity. To assess this, we performed pull down assays in cardiac homogenates, using a GST-G_M_ recombinant protein fragment (amino acids 1-778) (Figure 4B), which encompasses the region binding to both PP1 and PLN (amino acids 1-386 and 382-778, respectively). Western blot analysis revealed the presence of both PLN and PP1 in the GST-G_M_ sample but not in the GST control (Figure 4C), indicating the simultaneous association of G_M_ with PP1 and PLN.

To demonstrate the requirement of G_M_ as a targeting subunit of PP1 to its substrate, we performed another set of pull down experiments in cardiac homogenates, using full length GST-Inhibitor-1, the regulatory subunit that binds to PP1 and modulates its activity [12], [26]. Western blot analysis showed the presence of PP1 in the GST-Inhibitor-1 sample, supporting the occurrence of the PP1/Inhibitor-1 interaction. Importantly, the absence of PLN in the GST-Inhibitor-1 pull down sample (Figure 4C), suggested the requirement of G_M_ for effective targeting of PP1 to PLN. To further investigate this, we evaluated the association between MBP-PP1 and GST-PLN recombinant proteins in the absence or presence of G_M,_ using blot overlay assays. Western blots revealed the lack of binding between PP1 and PLN in the absence of G_M_, as the interaction of PP1 and PLN is not direct (Figure 4D). Conversely, protein binding was observed in the presence of G_M_ recombinant protein, which can bind directly and independently to both PP1 and PLN (Figure 4D). Collectively, these findings suggest the requirement of G_M_ in mediating the formation of the PP1/G_M_/PLN complex. In addition, they emphasize the existence of distinct PP1-mediated protein complexes with differential regulatory roles towards effective targeting and control of PP1 activity in cardiac muscle.

### Aberrant regulation of PP1 activity by a human inhibitor-1 variant (G147D)

**Figure 5:**
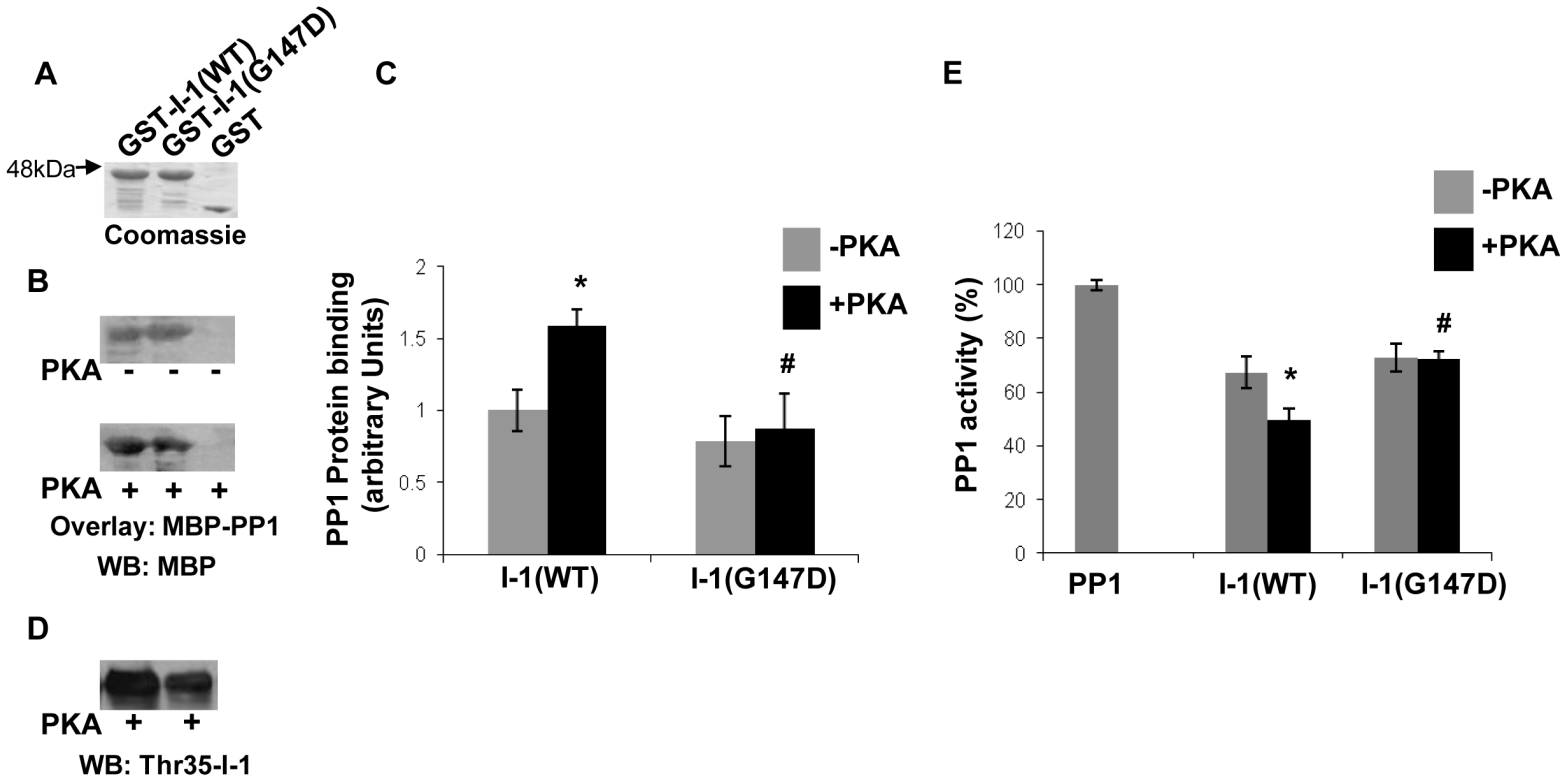
Inhibitor-1 variant (G147D) exhibits reduced binding and inhibition of PP1 upon PKA phosphorylation. (A) Coomassie blue stained gel of purified GST-Inhibitor-1 (I-1) recombinant proteins. (B) Blot overlay assay with MBP-PP1 protein and immunodetection with the MBP antibody determined similar levels of PP1 binding to both WT and G147D inhibitor-1 proteins under basal conditions. PKA phosphorylation resulted in significant enhancement of PP1's interaction with WT but no such effect was observed for G147D. (C) Quantification of PP1 binding to WT and G147D inhibitor-1 under basal and PKA conditions. n = 4; *t*-test, two-tailed, *P<0.05 vs WT (–PKA); #*P*<0.05 vs WT (+PKA). (D) Phosphorylation of G147D at Thr35 is reduced compared to WT. (E) PP1 activity measurements indicate impaired regulation of PP1 by G147D variant after PKA-phosphorylation. n = 4; *t*-test, two-tailed, *P<0.05 vs WT (–PKA); #*P*<0.05 vs WT (+PKA). WB: Western blot

While G_M_ ensures the correct targeting of PP1 to its substrate at the SR, other subunits of PP1 such as inhibitor-1 contribute towards the tight regulation of catalytic activity. The significance of inhibitor-1's regulatory role in the heart is indicated by the identification of a human polymorphism of inhibitor-1 (G147D) in heart failure patients [20]. Acute overexpression of this polymorphism in rat cardiomyocytes indicated that there was no effect on basal contractility, but the responses to β-adrenergic stimulation and phosphorylation of PLN were significantly decreased [20]. To better define the molecular mechanisms underlying this alteration, we investigated whether this human inhibitor-1 variant may be associated with aberrant PP1 regulation. For this analysis, we generated wild type (WT) or G147D mutant GST- inhibitor-1 recombinant proteins and used equal amounts of these proteins on blot overlay assays with MBP-PP1 protein (Figure 5A). There were no differences of WT or mutant inhibitor-1 binding to PP1 under basal conditions. However, upon PKA phosphorylation, WT inhibitor-1 showed significant enhancement of PP1 binding but the G147D variant did not exhibit any increases (Figure 5B-C, n = 4; *P*<0.05). Accordingly, both WT and mutant proteins inhibited PP1 to the same extent under basal conditions and only WT was able to enhance this inhibition upon PKA phosphorylation (Figure 5E, n = 4; *P*<0.05). Given that phosphorylation of inhibitor -1 at threonine 35 results in its activation as a potent inhibitor of PP1 [27], we next examined the phosphorylation levels of G147D recombinant protein. Western blot analysis using an antibody that specifically detects threonine 35-phosphorylated inhibitor-1 protein revealed a significant reduction in the phosphorylation levels of G147D variant, compared to WT (Figure 5D). These findings highlight the significance of inhibitor-1 phosphorylation in its interaction with PP1 and the subsequent inhibition of enzymatic activity.

### Impaired PP1 regulation by a human Hsp20 mutation identified in heart failure

**Figure 6:**
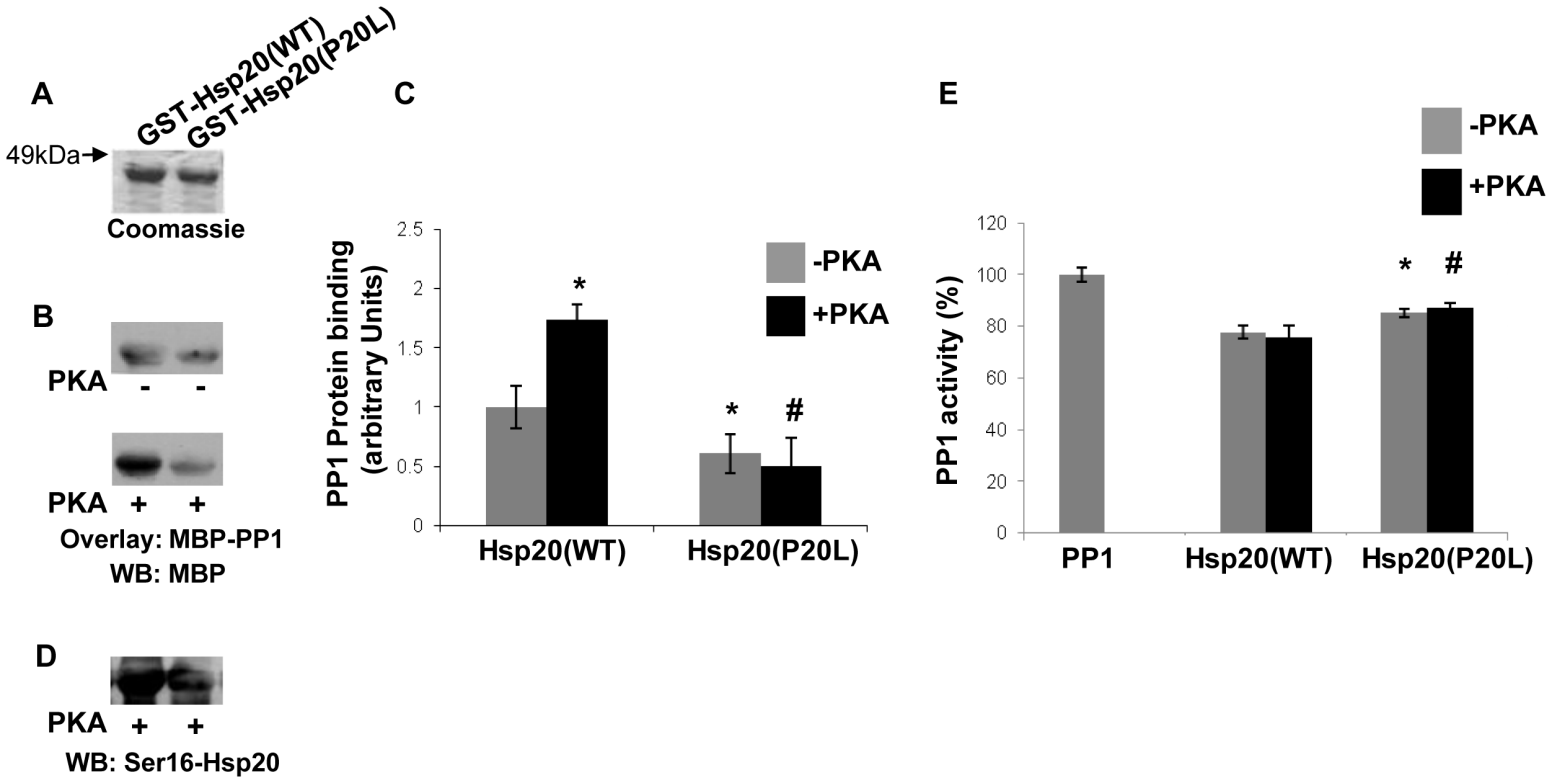
Hsp20 variant (P20L) variant exhibits diminished binding and inhibition of PP1. (A) SDS-gel stained with Coomassie blue showing purified GST-Hsp20 WT and P20L recombinant proteins. (B) Blot overlay assay with MBP-PP1 protein and immunodetection with MBP antibody demonstrates reduced PP1 binding to P20L protein. Phosphorylation of recombinant Hsp20 proteins by PKA treatment resulted in increased binding of PP1 to Hsp20-WT, however, this effect was not observed for P20L variant. (C) Quantification of PP1 protein binding to WT and P20L under basal and PKA conditions. n = 4; *t*-test, two-tailed, *P<0.05 vs WT (–PKA); #*P*<0.05 vs WT (+PKA). (D) Hsp20 variant P20L presents diminished phosphorylation levels at Ser16. (E) PP1 activity measurements showed reduced inhibition of PP1 by P20L. n = 4; *t*-test, two-tailed, *P<0.05 vs WT (–PKA); #*P*<0.05 vs WT (+PKA). WB: Western blot.

We have recently uncovered Hsp20 as an additional regulator of PP1 activity and PLN phosphorylation [13]. To further enhance our understanding of the significance of Hsp20 on the PP1-PLN axis, we evaluated the effects of wild-type Hsp20 and the human P20L-Hsp20 mutation on PP1 binding. The P20L mutation results in diminished phosphorylation of Hsp20 by PKA and abrogation of Hsp20 cardioprotective effects [21]. However, its effect on PP1 has not been evaluated. To address this, we generated WT and P20L GST-Hsp20 recombinant proteins (Figure 6A) and assessed PP1 binding under basal conditions (-PKA) or after PKA-phosphorylation (+PKA) of these Hsp20 proteins. Blot overlay assays with MBP-PP1 recombinant protein determined that, in contrast to WT, Hsp20-P20L exhibited significantly reduced binding to PP1 under basal conditions (Figure 6B-C, n = 4; *P*<0.05). Moreover, while PKA phosphorylation of Hsp20-WT resulted in significant enhancement in its interaction to PP1, no such effect was observed for the Hsp20-P20L mutant protein (Figure 6B-C, n = 4; *P*<0.05). Indeed, using an Hsp20 antibody that recognizes phosphorylation of residue serine 16, we observed significantly diminished phosphorylation levels of Hsp20-P20L, when compared to WT (Figure 6D), in accordance with our previous observations in adenovirally infected cardiomyocytes [21]. The results from the blot overlay assays were correlated with impaired regulation of PP1 activity since, in comparison to WT, the Hsp20-P20L variant was found to be a less potent inhibitor of PP1 (Figure 6E, n = 4; *P<0.05). These findings highlight the significance of Hsp20 as an interacting partner and regulator of PP1 and imply that the human P20L-mutant disrupts these properties.

## Discussion

The current study presents the first evidence of a multimeric protein complex regulating PP1 activity, which is associated with PLN and may impact cardiac physiology and pathophysiology. Our findings indicate that formation of this assembly is mediated through the direct and independent interactions of the targeting subunit G_M_ with both PP1 and PLN, facilitating compartmentalization of PP1 to its substrate. Moreover, efficient control of PP1 activity is achieved through its regulatory proteins, inhibitor-1 and Hsp20. Indeed, impaired association of PP1 with human genetic variants of inhibitor-1 or Hsp20 causes aberrant regulation of PP1 activity, which may contribute to cardiovascular dysfunction and heart disease. Furthermore, our biochemical analysis reveals that the multimeric protein complex controlling PP1 function is modulated by PKA phosphorylation, which causes the dissociation of PP1 from G_M_ and G_M_ from PLN. These findings point towards the regulatory nature of the PP1/G_M_/PLN interaction and suggest its significance in β-adrenergic responses of the heart.

PLN phosphorylation is of fundamental importance in the effective control of SERCA2 activity and cardiac contractility. Similarly, removal of the phosphate group resulting in PLN dephosphorylation represents another critical reaction during reversal of β-adrenergic response in the heart. This is mediated by PP1, the major isotype of serine/threonine phosphatases, the activity of which is under the tight control of auxiliary subunits. It has been estimated that nearly 200 different regulators govern the localization, substrate specificity and activity of PP1 [28], [29], [30]. Among these regulatory proteins, G_M_ has been isolated from SR membranes of striated muscle suggesting its involvement in targeting PP1 to the SR compartment [31]. This was rather unexpected given previous findings on the role of G_M_ as the subunit that targets PP1 to glycogen in skeletal muscle [32], [33]. Accordingly, a considerable number of studies have been focused on the role of G_M_ in glycogen metabolism of skeletal muscle, where it is involved in PP1 dephosphorylation of glycogen-metabolizing enzymes. G_M_ plays a central role in coordinating these reactions by forming protein complexes with glycogen, PP1, as well as glycogen synthase [34], [35]. The physiological significance of this regulatory function of G_M_ was demonstrated by the analysis of mouse models with G_M_ overexpression or ablation in skeletal muscles. These were associated with alterations in PP1 levels and activity, influencing glycogen synthase and impairing glycogen metabolism [36], [37], [38]. It was therefore proposed that dysfunction of the PP1/G_M_ axis may contribute to the pathophysiology of human type 2 diabetes [37]. Indeed, gene variants of *PPP1R3A*, the gene encoding G_M_, have been reported to cause impaired glycogen synthesis and muscle glycogen content resulting in insulin resistance and type 2 diabetes in human patients [39], [40], [41].

In contrast to skeletal muscle, the physiological significance of G_M_ in the heart has not been well established. An early report suggested its interaction with PLN [23], but that was not further explored. Our study is the first to provide direct evidence on the G_M_/PLN interaction and define the effect of PKA phosphorylation in modulating this association. In particular, we showed that the cytosolic regions of G_M_ and PLN interact and that G_M_ amino acids 382-778 are required for PLN binding. Our binding assays utilized a PLN construct, containing only the cytosolic portion of the protein, so the possibility of transmembrane binding, as previously hypothesized [23], may provide a second interaction between G_M_ and PLN, similar to the SERCA2/PLN interactions [42], [43]. Additionally, and in accordance with a previous study [44], we demonstrate that an N-terminal fragment of G_M_ that encompasses amino acids 1-386 is involved in its association with PP1. This may be attributed to the presence of the RVxF motif, a short stretch of an amino acid sequence, previously shown to be mediating the interaction of regulatory subunits with PP1 [44], [45], [46]. These findings reveal that different regions within the N-terminus of G_M_ are directly involved in its interactions with PLN and PP1, pointing to the essential role of G_M_ in coordinating formation of the PP1/PLN protein complex. It is therefore interesting to propose that G_M_ provides a protein scaffold, bringing together the catalytic enzyme and its substrate in order to achieve efficient and tight regulation of PLN dephosphorylation.

In contrast to targeting subunits such as G_M_ that do not affect PP1 activity, other regulatory proteins, including inhibitor-1 and Hsp20, modulate this enzyme with important implications in cardiac physiology. While the contribution of Hsp20 in Ca^2+^ cycling and cardiac function is now emerging [13], [47], inhibitor-1 has been extensively studied over the years [12], [26]. Interestingly, PKA-phosphorylation of inhibitor-1 or Hsp20 increases their binding to PP1 but in contrast to inhibitor-1, Hsp20 phosphorylation does not appear to influence further its inhibitory effect on PP1. While this effect was observed in an *in vitro* experimental system, it may reflect potential differences in inhibitory affinity or may suggest a different mode of regulation between these two PP1 inhibitory proteins. Importantly, the recent identification of human genetic variants in both inhibitor-1 and Hsp20 highlight their significance in cardiac pathophysiology [20], [21]. The G147D variant of inhibitor-1 has been associated with decreased phosphorylation of PLN and significant attenuation in β-adrenergic responses [20]. Our biochemical findings provide insights on the underlying mechanisms associated with these alterations and reveal that the G147D variant represents a less potent inhibitor of PP1 function. In particular, we showed that G147D exhibits diminished binding and inhibition of PP1 activity upon PKA phosphorylation. This may be due to reduced phosphorylation of threonine 35, a critical step required for activation of its inhibitory function [27]. Thus, impaired activation of the inhibitor-1 by the G147D variant may be associated with aberrant regulation of PP1 that consequently affects PLN phosphorylation and cardiac contractility. In addition to the G147D variant of inhibitor-1, detailed analysis of the Hsp20-P20L mutation provided further support on the significance of these inhibitory proteins in efficient regulation of PP1. Our findings establish that the Hsp20-P20L mutant exhibits impaired binding to PP1 and reduced inhibition of its activity. Decreased binding of P20L mutant to PP1, under both basal and PKA phosphorylation conditions, may be due to alterations in its secondary structure [21]. Collectively, the studies on these human variants highlight the importance of PP1 interactions with inhibitor-1 and Hsp20 in modulation of enzymatic activity with significant implications in cardiac function of the human carriers.

**Figure 7:**
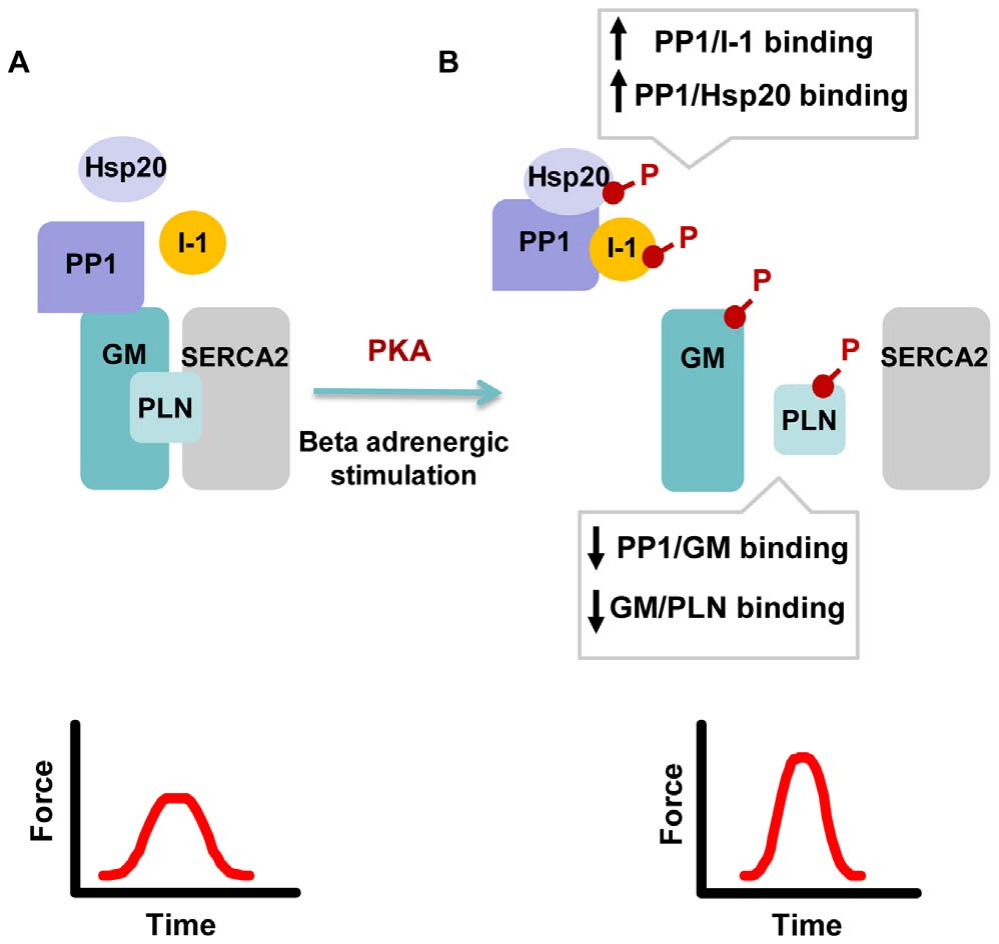
Schematic Representation of PP1 protein complexes regulating PLN phosphorylation and cardiac contractility. A regulatory PP1 signaling complex controls PLN phosphorylation. (A) Under basal conditions, G_M_ associates with both PP1 and PLN, enabling effective targeting of the phosphatase to its substrate. (B) Following β-adrenergic stimulation, protein phosphorylation leads to dissociation of the PP1/G_M_/PLN complex, enabling PP1 to bind with high affinity to its regulatory proteins Inhibitor-1 and Hsp20. This results in inhibition of PP1 and enhanced phosphorylation of PLN as well as increased cardiac contractility.

β-adrenergic stimulation of the heart leads to activation of PKA and subsequent phosphorylation of a number of proteins [12], [26], [48]. Our findings support the notion that part of the PKA regulatory effects include PP1 inhibition through a protein complex formation. Specifically, PKA phosphorylation influences protein associations, including interactions of PP1 with its targeting subunit G_M_ [44], [49] and inhibitor-1, as well as the association between G_M_ and PLN. Based on these *in vitro* findings, we propose the dynamic existence of a highly regulatory signaling complex that controls PLN phosphorylation and cardiac function (Figure 7). Under basal conditions, PP1 associates with G_M_ to enable targeting of the phosphatase to PLN at the SR. Following β-adrenergic stimulation, protein phosphorylation leads to dissociation of G_M_ from both PLN and PP1, without affecting enzymatic activity [33], [49]. Instead, control of the catalytic subunit's activity is achieved by the subsequent association with high affinity to its regulatory proteins, ultimately leading to PP1 inhibition and restoration of basal conditions. The dynamic and transient nature of these interactions may be of physiological significance during the heart's response to β-adrenergic stimulation, by enabling cardiac muscle to rapidly increase output demands. Accumulating evidence has recently suggested the involvement of other components, including A-kinase anchoring proteins (AKAPs) and phosphodiesterases (PDEs) to ensure compartmentalization of PKA signaling in order to promote rapid and efficient sub-cellular regulation [50], [51], [52], [53], [54]. Given the contribution of Ca^2+^ homeostasis in heart failure, components of this system may represent promising therapeutic targets [55]. Indeed, a recent study has shown that overexpression of active inhibitor-1 preserves cardiac function following *in vivo* gene transfer in a porcine model of heart failure [56]. Similarly, down-regulation of PP1 was shown to be beneficial in preventing heart failure progression in a cardiomyopathy mouse model [57]. Future studies will delineate the potential of modulating other components in this regulatory complex that controls Ca-cycling and contractility in the heart.
